# Dietary inflammatory index, and depression and mortality risk associations in U.S. adults, with a special focus on cancer survivors

**DOI:** 10.3389/fnut.2022.1034323

**Published:** 2022-12-14

**Authors:** Yuzheng Zhang, Yanhua Wu, Yangyu Zhang, Donghui Cao, Hua He, Xueyuan Cao, Yuehui Wang, Zhifang Jia, Jing Jiang

**Affiliations:** ^1^Division of Clinical Epidemiology, The First Hospital of Jilin University, Changchun, China; ^2^Department of Epidemiology and Biostatistics, School of Public Health, Jilin University, Changchun, China; ^3^Department of Gastric and Colorectal Surgery, General Surgery Center, The First Hospital of Jilin University, Changchun, China; ^4^Department of Geriatrics, The First Hospital of Jilin University, Changchun, China

**Keywords:** depression, inflammation, mortality, cancer survivor, dietary inflammatory index (DII)

## Abstract

**Introduction:**

A higher risk for depression and mortality is associated with the inflammatory potential of diet measured through the Dietary Inflammatory Index (DII). The roles of DII in the risk of depression and death in cancer survivors were unclear. We aimed to examine the association between energy-adjusted DII (E-DII) score and risk of depression, and mortality using data from the 2007–2018 National Health and Nutrition Examination Survey (NHANES), with a special focus on cancer survivors.

**Methods:**

The 24-h dietary recall interview was used as a basis to calculate the E-DII score and the Patient Health Questionnaire-9 (PHQ-9) was used to measure the depressive outcomes. Logistic regression analyses were performed to determine the association between quartiles of E-DII score and depression. Cox proportional hazard regression and competing risk analyses were used to estimate the risks of quartiles of E-DII score or depression on mortality.

**Results:**

A total of 27,447 participants were included; including 24,694 subjects without cancer and 2,753 cancer survivors. The E-DII score and depression were not distributed differently between the two groups. However, the E-DII scores were positively associated with within each group’s depression (all *P* trend < 0.001) and participants with higher E-DII scores had a higher risk of depression (subjects without cancer: OR_Q4__vs__Q1_: 2.17, 95% CI: 1.75–2.70; cancer survivors: OR_Q4__vsQ1_: 1.78, 95% CI: 1.09–2.92). The median follow-up time were 87 person-months, a total of 1,701 (4.8%) and 570 (15.2%) all-cause deaths in subjects without cancer and cancer survivors were identified by the end of 2019. The highest E-DII scores quartile was associated with the highest risk of all-cause (HR_Q4__vsQ1_: 1.90, 95% CI: 1.54–2.35) and cardiovascular disease (CVD) cause death (HR_Q4__vsQ1_: 2.50, 95% CI: 1.69–2.3.7) in the subjects without cancer. Moreover, participants with depressive symptoms had higher all-cause mortality (HR: 1.29, 95% CI: 1.04–1.59). No significant correlation was found for E-DII scores or depression with all-cause, cancer-cause or CVD-cause mortality in cancer survivors.

**Conclusion:**

Our findings demonstrate that E-DII score was positively associated with depression risk. A higher E-DII score or depressive symptom may increase the risks of all-cause and CVD-cause mortality only among general subjects.

## 1 Introduction

Diet plays a key, but neglected role, for health as a modifiable factor. Unhealthy dietary patterns characterized by higher energy consumption, fat and sugar products, red and processed meats, and alcohol, which may influence health, and associated with an increased risk of adverse outcomes (1, 2). The dietary pattern effect on adverse health outcomes is partly through its influence on inflammatory pathways (3). Dietary Inflammatory Index (DII) is developed to characterize the overall inflammatory potential of an individual’s diet (4) and a higher DII score represents a pro-inflammatory diet. In terms of mental health, DII score is positively associated with depression symptoms risks in limited populations, such as patients with chronic diseases, female nurses, elderly over 55 years or U.S. adults based on only three cycles (2007–2012) of National Health and Nutrition Examination Survey (NHANES) (5–8). A higher DII score was additionally observed to be related with higher risks of various chronic inflammation-related health outcomes including cancer and cardiovascular disease (CVD) incidence and related mortality (9).

The U.S. cancer survivor population is projected to expand to 22.1 million by 2030 with a cancer incidence increment and treatment advancements (10). Cancer survivors are more likely to suffer common mental disorders such as depression (11, 12). Depressive symptoms reduce cancer survivors’ quality of life and treatment compliance, promote disease progression, and increase mortality risk (13–15). A previous study found that cancer survivors have poor dietary quality compared with subjects without cancer (16); however, a few studies investigate the dietary effect of diet quality in depressive symptoms. Further evidence is required to support evidence-based preventive interventions that will improve mental health and survival among cancer survivors.

The association between energy-adjusted DII (E-DII) score and depression is unclear in U.S. adults, especially in cancer survivors although E-DII score is positively associated with depression in limited populations (5–8). Fewer still have evaluated whether depression affected the relationship between E-DII score and mortality. Therefore, this study was performed using the data in a large prospective cohort of a nationally representative population, the 2007–2018 National Health and Nutrition Examination Survey (NHANES) database, aimed to (1) assess the association between energy-adjusted DII (E-DII) score and depression risk and (2) explore the E-DII score and depression on mortality risk impact in subjects without cancer as well as cancer survivors.

## 2 Materials and methods

### 2.1 Study design and participants

NHANES project uses a complex, multistage, probability based, cross-sectional design to select a representative sample of the U.S. population every 2 years to assess the health and nutritional status in the U.S. NHANES’ methods and protocols are described in more detail previously (17). The National Center for Health Statistics Institutional Review Board approved this survey and each participant signed the informed consent.^1^

The current study included participants that had completed NHANES’ Questionnaire in the 2007–2018 cycles (*n* = 57,381). Participants who were (1) aged under 20 (*n* = 22,612), as only participants aged 20 or more needed to answer the question on previous cancer, (2) lack of information on diet (*n* = 3,980), previous cancer (*n* = 24), or depression screening (*n* = 2,077), (3) total energy intake outside the predefined range of 800–4,200 kcal/d for men or 600–3,500 kcal/d for women (18) (*n* = 1,241) were excluded from further analysis.

### 2.2 Cancer variables

Participants aged 20 or older needed to answer the question “Have you ever been told by a doctor or other health professional that you had cancer or a malignancy of any kind?” according to the design of NHANES. Individuals who responded “yes” was defined as a cancer survivor and who responded “no” was defined as no cancer participant. Participants who responded “yes” further answered the question “What kind of cancer was it, and how old were you when cancer was diagnosed?” to collect the cancer sites and numbers. Up to three sites were recorded and the number was grouped into two categories: 1 and ≥2 times. The cancer sites were further classified as digestive system tumors, if there was only one recording including colon, rectum, esophagus, gallbladder, liver, pancreas, and stomach were reported and other tumors (19). Cancer duration was computed by subtracting the age the first cancer was diagnosed from the screening age (20), and the duration was divided into two categories: ≤5 years and >5 years.

### *2.3* Dietary inflammatory index

Shivappa et al. reported the DII score, and development and validation in diet. They summarized the inflammatory effect score of 45 kinds of nutrients and estimated the global means with standard deviations for each nutrient combining results from 11 populations around the world (4). This DII score could be used wholly or partly of the 45 kinds of nutrients, as when the number of nutrients applied for the calculation of DII is <30, the DII score is still valid (21, 22). NHANES project uses a 24-h dietary recall to measure dietary information, which contains 27 of the above 45 food nutrients. The following nutrients were used to calculate the E-DII score: carbohydrates, protein, total fat, fiber, cholesterol, saturated fatty acids, monounsaturated fatty acids, polyunsaturated fatty acids, β-carotene, vitamin A/B1/B2/B6/B12/C/D/E, folic acid, iron, magnesium, zinc, selenium, omega-3, and omega-6 polyunsaturated fatty acids, alcohol, and caffeine. A *Z*-score is first computed for each participant for each food parameter by subtracting the world mean of this food from each participant’s estimated intake and then divided by the world standard deviation. The Z-score was then converted to percentile rank and centered by doubling the value and subtracting 1. Finally, DII score was obtained by summing the products of the inflammatory effect score and the centered percentile of each food parameter. The E-DII scores were calculated based on the intake of dietary components as expressed per 1,000 kcal consumed given the fact that total energy requirements are related to body size, metabolic efficiency, and physical activity, and then repeating an analogous DII calculation process (23). E-DII score was calculated in participants completing the dietary questionnaire in present study (*n* = 27,447). The E-DII score ranged from -5.54 to 5.55, and discretized by the quartiles.

### 2.4 Depression outcomes

The primary outcome of interest was a symptomatology of depression, as measured by the Patient Health Questionnaire-9 (PHQ-9). The PHQ-9 is a 9-item module from the full PHQ and has been validated against mental health professional interviews (24, 25). Each item is scored 0–3 and the PHQ-9 score for each participant ranges from 0 to 27. Higher values represent more severe depressive symptomatology. A score ≥ 10 has a sensitivity of 88% and specificity of 88% in predicting major depression (24, 25). The participants in this study were grouped into no depression (PHQ-9 < 10) and depression (PHQ-9 ≥ 10) based on this cut-off value.

### 2.5 Ascertainment of mortality

The NCHS database provides the NHANES public-use linked mortality files until December 31, 2019, which was linked to the National Death Index (26). The survival time (months) was defined as the duration from the date of interview in NHANES to the date of death. The survival time was right-censored and calculated by subtracting the date of survey participation from the end of the follow-up (December 31, 2019) for survivor participants.

### 2.6 Socio-demographic covariates

Potential confounders were considered, including age, gender, race/ethnicity, body mass index (BMI), tobacco use, alcohol use, co-morbidity index, educational level, marital status, and health insurance. Age was categorized into two classes as 20–64 years and >65 years. Race/ethnicity was examined in two race/ethnicity groups: non-Hispanic white and others. BMI was described as underweight or normal (≤24.9 kg/m^2^), overweight (25–29.9 kg/m^2^), and obese (≥30 kg/m^2^). Tobacco use was coded as “Yes” for smoked at least 100 cigarettes in life and “No” for non-smokers (27). The cut-off for “No” and “Yes” was at least 12 alcohol drinks in the past year for alcohol use (28). Co-morbid conditions consisted of hypertension, diabetes, failing or weak kidneys, asthma, coronary heart disease, congestive heart failure, heart attack, stroke, emphysema, chronic bronchitis, or thyroid problems. The co-morbidity index was divided into four groups (0, 1, 2, ≥3) according to the total number of reported conditions (29). Participants’ education level was grouped into two categories, “<High school” and “≥High school.” Marital status was categorized as never married, widowed/divorced/separated, and married/living with partner. Health insurance status was defined as “No” for no health insurance and “Yes” for having any kind of health insurance.

### 2.7 Statistical analyses

The NHANES provides the weight for each participant to account for the design, non-response, and post-stratification adjustment of the survey to represent the US total population. The 12-year sample weights were calculated to be adjusted for the difference between the six cycles provided by NHANES and then weighted populations were used for analysis of DII and depression (30). The subjects’ characteristics were described as unweighted counts with weighted percentages, and compared using χ^2^ test or Cochrane-Armitage trend test. Odds ratios (ORs) with their 95% confidence intervals (CIs) were computed by multivariate logistic regression model to assess the predictive E-DII score value on depression after adjusting for social-demographical covariates. Subgroup analysis were performed according to the previous cancer diagnosis times, primary site, and the duration of cancer diagnosis. Hazard ratios (HRs) and 95% CIs were estimated using multivariate Cox proportional hazard regression model to evaluate the role of E-DII score or depression in mortality. Competing risk models were performed to evaluate the association between E-DII score or depression and the risk of the cancer-cause or CVD-cause death with the competing risk category referred to non-cancer-cause death or non-CVD-cause death considering that the competitive-risk model could better reflect the real death risk of cancer and CVD, which are the main causes of death in NHANES. The possible interaction between E-DII and depression was examined by including the product term in the Cox proportional hazard regression model and competing risk model. The SPSS software (version: 24.0, SPSS, Chicago, IL, USA) and Stata software (version: 15.0, College Station, TX, USA) was used to conduct all analyses and the differences were considered statistically significant at a two-tailed value of *P* < 0.05.

## 3 Results

**FIGURE 1:**
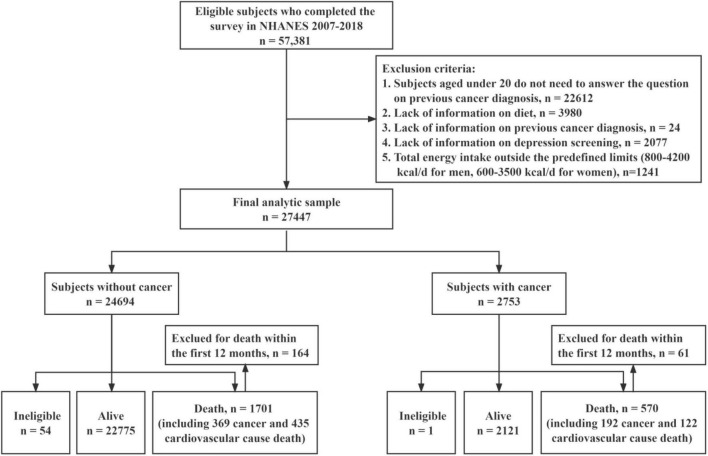
Flow chart of participant selection.

A total of 27,447 participants who met the criteria were included in the analysis, including 24,694 subjects without cancer and 2,753 cancer survivors (Figure 1). Table 1 shows the subjects’ baseline characteristics. Cancer survivors were more likely to be aged ≥65 years (51.1 vs 15.2%), female (56.3 vs 51.5%), non-Hispanic white (86.1 vs 65.7%), tobacco user (53.3 vs 43.1%), more than high-school educated (67.9 vs 61.4%), and have health insurance (94.9 vs 82.0%) compared to subjects without cancer. Cancer survivors were less likely to be without chronic comorbidity (26.5 vs 50.7%) and never married (5.9 vs 19.4%). There was no significant difference between subjects without cancer and cancer survivors for the distribution of E-DII score and depression.

**TABLE 1:** Population characteristics by cancer status in NHANES 2007–2018.

Characteristic^1^	Subjects without cancer (*n* = 24,694)	Subjects with cancer (*n* = 2,753)	*P* value
**Age (years, *n*, %)**			
<65	19,708(84.8)	1,104(48.9)	< 0.001
≥65	4,986(15.2)	1,649(51.1)	
**Gender (*n*, %)**			
Male	12,005(48.5)	1,308(43.7)	< 0.001
Female	12,689(51.5)	1,445(56.3)	
**Race/Ethnicity (*n*, %)**			
Non-Hispanic white	9,817(65.7)	1,843(86.1)	< 0.001
Other or multi-race/ethnicity	14,877(34.6)	910 (13.9)	
**BMI (kg/m^2^, *n*, %)**			
<24.9	6,813(28.9)	698 (26.8)	0.210
25–29.9	8,031(32.8)	956 (34.1)	
≥30	9,658(38.3)	1,067(39.1)	
**Tobacco use (*n*, %)**			
No	14,013(56.9)	1,254(46.7)	< 0.001
Yes	10,671(43.1)	1,498(53.3)	
**Alcohol use (*n*, %)**			
No	5,815(22.6)	669 (24.1)	0.219
Yes	14,972(77.4)	1,618(75.9)	
**Co-morbidity index (times, *n*, %)**			
0	11,710(50.7)	597 (26.5)	< 0.001
1	7,077(28.9)	857 (31.8)	
2	3,364(12.3)	620 (21.1)	
≥3	2,543(8.0)	679 (20.5)	
**Education (*n*, %)**			
<High school	11,513(38.6)	1,153(32.1)	< 0.001
≥High school	13,163(61.4)	1,599(67.9)	
**Marital status (*n*, %)**			
Never married	4,786(19.4)	176 (5.9)	< 0.001
Widowed/Divorced/Separated	5,195(17.1)	915 (27.6)	
Married/Living with partner	14,701(63.5)	1,660(66.5)	
**Health insurance (*n*, %)**			
No	5,493(18.0)	161 (5.1)	< 0.001
Yes	19,173(82.0)	2,587(94.9)	
**E-DII score quartile**			
Q1	5,483(24.8)	692 (26.8)	0.174
Q2	5,997(25.1)	632 (24.3)	
Q3	6,375(24.9)	711 (25.7)	
Q4	6,839(25.2)	718 (23.2)	
**Depression status (*n*, %)**			
No depression	22,509(92.2)	2,471(91.8)	0.473
Depression	2,185(7.8)	282 (8.2)	
**Final mortality status (*n*, %)**			
Alive	22,775(95.2)	2,121(84.8)	< 0.001
All-cause death	1,701(4.8)	570 (15.2)	

^1^The characteristics of the subjects were described as unweighted counts with weighted percentages.

We further evaluated the association of E-DII scores with depression by comparing the proportion of depression in the four quartiles of E-DII scores in subjects without cancer and cancer survivors, respectively (Table 2). The depression proportion increased as the E-DII quartile climbed, both in subjects without cancer and cancer survivors. The proportion of depression tripled from 3.9% in Q1 to 11.9% in Q4 in subjects without cancer (*P* trend < 0.001), and from 5.7% in Q1 to 15.2% in Q4 in cancer survivors (*P* trend < 0.001). The E-DII scores association with depression were assessed further by adjusting for potential confounders as several variables were found to be related to E-DII score depression (Supplementary Table 1) (5, 31). The ORs of having depression across the quartiles of E-DII scores are shown in Table 2. There was a significant upward trend in the odds of having depression with the increment of E-DII scores (*P* trend < 0.001), subjects in the Q4 of E-DII score were found to be at an increased risk of developing depression compared with those in Q1 (OR: 2.17, 95% CI: 1.75–2.70) for subjects without cancer, after adjusting for variables including age, gender, race/ethnicity, BMI, tobacco use, co-morbidity index, education level, marital status, and health insurance status. The significant upward associations were also observed after adjusting for the same covariate (*P* trend < 0.001) and the highest quartile of the E-DII score had highest risk of depression than those in the lowest quartile (OR: 1.78, 95% CI: 1.09–2.92) for cancer survivors. Subgroup analyses were done and classified by previous cancer diagnosis times, primary cancer site, and duration of diagnosis in cancer survivors to explore the association between E-DII score and depression (Supplementary Table 2). The magnitudes of associations were similar across the subgroups.

**TABLE 2:** Association between dietary inflammatory index score and depression status across cancer status and cancer related variables in NHANES 2007–2018.

		Dietary inflammatory index score (quartiles)								*P* trend
				
		Q1		Q2		Q3		Q4		
**Subjects without cancer**										
Depression^1^ (*n*, %)		266 (3.9)		471 (6.7)		575 (8.6)		873 (11.9)		< 0.001
Crude OR (95% CI)		Ref		1.80(1.43,2.27)		2.33(1.94,2.80)		3.35(2.81,3.99)		< 0.001
Adjusted OR^2^ (95% CI)		Ref		1.61(1.26,2.06)		1.86(1.49,2.32)		2.17(1.75,2.70)		< 0.001
**Subjects with cancer**										
Depression^1^ (*n*, %)		44 (5.7)		43 (4.9)		67 (7.7)		128 (15.2)		< 0.001
Crude OR (95% CI)		Ref		0.84(0.48,1.47)		1.37(0.78,2.40)		2.95(1.83,4.73)		< 0.001
Adjusted OR^2^ (95% CI)		Ref		0.80(0.43,1.51)		1.00(0.52,1.92)		1.78(1.09,2.92)		0.019

^1^The distribution of depression were described as unweighted counts with weighted percentages.

^2^Adjusted OR: adjusted for age, gender, race/ethnicity, BMI, tobacco use, co-morbidity index, education level, marital status, and health insurance status.

We finally evaluated the roles of E-DII score or depression on the risks of death of all-cause, and cancer-cause or CVD-cause in subjects without cancer and cancer survivors. A total of 2,496 deaths were identified and 225 deaths within the first 12 months of follow-up, 164 in subjects without cancer and 61 in cancer survivors, were excluded to reduce the possibility of reverse causation during a median follow-up time of 87 person-months (32). The subjects without cancer had 1,701 all-cause deaths, including 369 (22.7%) cancer deaths and 435 (24.4%) CVD deaths, while cancer survivors had 570 all-cause deaths, including 192 (33.9%) cancer deaths and 122 (22.3%) CVD deaths. As expected, the all-cause, cancer-cause, and CVD-cause deaths were higher in cancer survivors than in subjects without cancer (15.2 vs 4.8%, 5.2 vs 1.1%, 3.4 vs 1.2%). A significantly higher risk of all-cause (HR:1.90, 95%CI: 1.54–2.35) were observed for subjects with Q4 of E-DII score compared to those of Q1 in subjects without cancer after adjusting for the effects of depression and other variables related to survival, and similar association was also observed between E-DII and CVD-cause death (HR_Q4VS Q1_: 2.21, 95%CI: 1.44–3.39) after considering the competitive risk of other causes of death as shown in Table 3. Meanwhile, depression was associated with a higher risk of all-cause mortality (HR: 1.29, 95% CI: 1.04–1.59) after accounting for the E-DII score influence and other variables, but not with cancer-cause or CVD-cause death in the multivariable-adjusted competing risk regression models. No significant correlations were found for E-DII score or depression with all-cause, cancer-cause, or CVD-cause death (Table 3) in cancer survivors.

**TABLE 3:** Association of dietary inflammatory index score and depression status with all-cause, cancer-cause and cardiovascular disease (CVD)-cause death in NHANES 2007–2018.

	Dietary inflammatory index score (quartiles)				*P* trend	Depression status		*P*
	Q1	Q2	Q3	Q4		No depression	Depression	
**All-cause death**								
**Subjects without cancer**								
All-cause death^1^ (*n*, %)	273 (3.2)	402 (4.5)	448 (5.3)	578 (6.4)	< 0.001	1,509 (4.6)	192 (7.0)	< 0.001
Crude HR (95%CI)	Ref	1.43(1.15,1.76)	1.67(1.36,2.06)	2.02(1.66,2.45)	< 0.001	Ref	1.52(1.24,1.85)	< 0.001
Adjusted HR^2^ (95%CI)	Ref	1.40(1.16,1.70)	1.51(1.22,1.87)	1.90(1.54,2.35)	< 0.001	Ref	1.29(1.04,1.59)	< 0.001
**Subjects with cancer**								
All-cause death^1^ (*n*, %)	144 (16.6)	142 (14.8)	146 (13.8)	138 (15.5)	0.605	524 (15.4)	46 (13.4)	0.421
Crude HR (95%CI)	Ref	1.04(0.77,1.41)	0.88 (0.65.1.20)	1.02(0.75,1.37)	0.803	Ref	0.85(0.57,1.24)	0.381
**Cancer-cause death**								
**Subjects without cancer**								
Cancer-cause death^1^ (*n*, %)	73 (0.9)	81 (1.0)	100 (1.4)	115 (1.3)	0.088	342 (1.1)	27 (1.1)	0.874
Crude HR (95%CI)	Ref	1.09(0.72,1.63)	1.49(1.00,2.21)	1.45(0.98,2.13)	0.016	Ref	0.93(0.54,1.58)	0.782
Adjusted HR^2^ (95%CI)	Ref	1.00(0.73,1.38)	1.09(0.80,1.49)	1.13(0.83,1.56)	0.357	Ref	0.91(0.52,1.58)	0.728
**Subjects with cancer**								
Cancer-cause death^1^ (*n*, %)	53 (7.0)	39 (4.6)	53 (5.1)	47 (6.1)	0.615	175 (5.8)	17 (4.8)	0.561
Crude (95%CI)	Ref	0.85(0.56,1.29)	1.03(0.70,1.50)	0.94(0.64,1.39)	0.969	Ref	0.88(0.54,1.45)	0.625
**CVD-cause death**								
**Subjects without cancer**								
Cancer-cause death^1^ (*n*, %)	57 (0.6)	109 (1.3)	120 (1.3)	149 (1.7)	< 0.001	382 (1.2)	53 (1.9)	0.009
Crude HR (95%CI)	Ref	2.05(1.39,3.07)	2.03(1.37,3.01)	2.59(1.76,3.01)	< 0.001	Ref	1.61(1.23,2.30)	0.009
Adjusted HR^2^ (95%CI)	Ref	1.91(1.25,2.92)	1.72(1.12,2.63)	2.21(1.44,3.39)	0.001	Ref	1.35(0.92,1.98)	0.127
**Subjects with cancer**								
Cancer-cause death^1^ (*n*, %)	36 (4.6)	37 (4.8)	27 (3.6)	22 (2.2)	0.039	115 (4.0)	7 (2.4)	0.242
Crude (95%CI)	Ref	1.22(0.77,1.93)	0.77(0.47,1.26)	0.65(0.38,1.10)	0.036	Ref	0.55(0.26,1.19)	0.128

^1^The distribution of mortality were described as unweighted counts with weighted percentages.

^2^Adjusted HR adjusted for age, gender, race/ethnicity, BMI, tobacco use, co-morbidity index, education level, marital status, health insurance status and depression or E-DII score.

## 4 Discussion

This study examined the E-DII score relationship with depression and depression with mortality using data from NHANES 2007–2018, with special focus on cancer survivors. Our results demonstrate that subjects with a higher E-DII score had a higher depression risk, both in subjects without cancer or in cancer survivors. However, the pro-inflammatory diet effect characterized by higher E-DII scores and depression on survival was only observed in the subjects without cancer.

Our results are consisted with several community-based studies (5, 31, 33). We examined the association within the subjects without cancer and cancer survivors, respectively, using the data of NHANES 2007–2018. Results from the two groups both show a linear upward trend that the proportion of depression increases with the E-DII increment. A previous study only included NHANES 2007–2012 adult participants reported similar associations between DII scores without energy adjustment and depressive symptoms (OR_Q5VS Q1_:2.26, 95%CI: 1.60–3.20) (8). These studies indicated that the relationship between dietary inflammation and depression was robust. In the current study, the median E-DII scores for the most pro-inflammatory quartile in in NHANES 2007–2012 and NHANES 2013–2018 were 2.1 and 2.2, respectively, whereas the upper quartile median E-DII score of was 0.6 in a previous study based on the Mediterranean diet (34), indicating that the dietary inflammation potential pattern of U.S. adults was higher and the secular E-DII score trend was consistent. There are currently no studies that explore the relationship between DII score and depression among cancer survivors. A similar association between E-DII score and depression was also observed in cancer survivors. A possible explanation is that insufficient nutrient intake is common in cancer survivors, such as digestive tumor (35, 36), which weakens the depressive symptoms caused by pro-inflammatory or anti-inflammatory diet by affecting the level of systemic inflammation. Several studies reported that the majority of survivors modified their diet intake after their cancer diagnosis (37–39) and changing unhealthy dietary patterns has been shown to lessen cancer treatment sequelae, possibly reducing the risk of recurrence for specific cancer types (40). The E-DII distribution score were not statistically significant between subjects without cancer and cancer survivors; however, we did observe a gradually decreased proportion from the most anti-inflammatory quartile to the most pro-inflammatory quartile. This little shift suggests that cancer survivors may change dietary patterns post cancer diagnosis, but this remains subject to speculation. Prospective research is warranted to explore the relationships between dietary changes and depressive symptoms in the future.

The mechanism between the positive association between DII score and depression is not clearly clarified. Cumulating systemic inflammatory level after consuming the pro-inflammatory diet may underlie the depression (21). Several evidences could explain the underlying association between systemic inflammation and depression, at least partly. First, systemic inflammation can produce overdose free radicals resulting in oxidative damage (41). The oxidative stress biomarkers are increased in depressive patients (42). A likely explain is that the brain is particularly susceptible to oxidative damage because of its high lipid content and energy consumption and relatively limited anti-oxidants defense (43). Second, systemic inflammation can increase the level of proinflammatory cytokines released from immune cells (44). Proinflammatory cytokines may induce the hyperactivity of the hypothalamus–pituitary–adrenal axis and an imbalance of tryptophan metabolism, which are all involved in the etiology of depression (45). Lastly, systemic inflammation may influence the brain-gut-microbiota axis, the intestinal microbiota and diet play an essential role in these gut-brain interactions and be involved in the pathogenesis of depression (46).

Previous studies reported higher E-DII scores and predict a higher risk of adverse health outcomes, including all-cause, cancer-cause, and CVD-cause mortality (47–51). Our study also observed the positive E-DII score association with all-cause and CVD cause mortality in subjects without cancer, but not associated with cancer-cause death. However, in cancer survivor subjects, reports on the correlation between E-DII score and mortality among different studies were controversial. The two studies, with median 13.3 and 14.6 years of follow-up, deemed that higher E-DII were associated with a shorter overall survival time in survivors of breast cancer (52, 53) while another study on colorectal cancer survivors (54),with a median follow-up time of 7 years, found no E-DII association with cancer prognosis. The inconsistency may result from a variety of cancer types with a different mortality and a diverse follow-up time. There are about 27.8 and 27.0% deaths of breast cancer survivors (52, 53), and about 15% deaths of colorectal cancer survivors (54). However, despite the median follow-up time being over 7 years in our study, only 4.8% of deaths in subjects without cancer and 15.2% of deaths in cancer survivors in our study. Further studies with extended follow-up time and sample size are warranted in the future. In the current study, we compared the subjects without cancer and cancer survivors who have a higher risk of death (HR: 3.59, 95%CI: 3.16–4.07), and cancer survivors had a higher co-morbidity index and are more likely to be tobacco users, which enhance the death risk and mask the relatively weak contribution of E-DII scores on mortality.

Several theories may explain the underlying mechanism between higher E-DII diets and mortality risk. High-DII diets accelerate the rate of telomere shortening, and shorter telomeres indicate increased mortality risk in the general population (55). Diets with high E-DII scores additionally contribute to increased levels of inflammatory cytokines, including C-reactive protein, interleukin 6, and tumor necrosis factor-α, which are associated with a higher chronic diseases and mortality risk (56, 57). The increased inflammatory cytokine level partly account for the mechanisms through high-DII diets increases the CVD-cause death risk. Cumulated inflammatory cytokines cause attraction and migration of inflammatory cells into vascular tissue (58), and enhance the cellular adhesion molecules expression, such as selectins and cadherins, which mediate white blood cell adhesion to the vascular endothelium (59). Moreover, cumulative inflammatory level produce overdose free radicals that cause oxidative damage (60), which are associated with CVD risk and mortality (61). Nonetheless, the exact mechanism needs further confirmation.

As expected, we also observed the association of depression with all-cause mortality in subjects without cancer after E-DII score adjustment and other covariates. Depression itself increases mortality (62) and depressive victims also suffer from other comorbidities such as asthma and CVD, which could worsen the overall health status and enhance the risk of mortality (63, 64). In addition, there were no significant interaction effects between E-DII score and depression in their association with all-cause, cancer-cause death, and CVD-cause death (Supplementary Table 3). These independent associations of E-DII score or depression with mortality suggest that their pathogenesis on mortality may differ and further studies are warranted.

Our study has strengths. We conducted the present analysis using the public data from NHANES, a nationally representative survey with high-quality measurements, which improves the generalizability of the results. Additionally, this is the first study to explore the relationship between E-DII score and depression among cancer survivors using NHANES, and this work also examine for the first time the association between the E-DII score, depression and mortality. Previous researchers have suggested that dietary supplementation with nutrients such as vitamin (65, 66), minerals (67) and polyunsaturated fatty acids (68, 69) could alleviate inflammation-related depression. The emerging field of nutritional epidemiology turns its attention to the relationship between dietary patterns and health outcomes, rather than focusing on specific nutrients or foods. The use of E-DII as an indicator to directly and reasonably connect the three of nutrition, inflammation, depression and mortality, may have clinical and public health significance for promoting good mental health and reducing death induced by diet. However, we acknowledge some limitations in this study. First, estimation of E-DII score effect on depression is based on data from cross-sectional design. This may limit the evidence grade on causal inference. Second, the E-DII score and depression score are calculated by self-report from 24 h dietary information and PHQ-9, which could not preclude the possibility of information biases. However, data on depression and dietary patterns were based on repeated and validated records and this could minimize this concern. Third, cancer-related measures such as tumor stage and therapy were not taken into account because of no relevant data. We adjusted for potential predictors of survival to minimize the potential effects of confounding factors. Fourth, reverse causation could be possible, as dietary consumption and nutritional status may be altered when people feel unwell. Future studies with high quality design are required to confirm the effect of the dietary inflammatory score on depression and mortality risk.

## 5 Conclusion

In summary, we have demonstrated that higher E-DII score is associated with an increased depression risk. Higher E-DII score and depressive symptoms are related to increased risks of all-cause mortality or CVD-cause mortality in subjects without cancer, but not in cancer survivors. Encouraging anti-inflammatory diet may be an effective way to prevent depression and reduce mortality risk may have clinical and public health implications.

## Data availability statement

Publicly available datasets were analyzed in this study. This data can be found here: https://wwwn.cdc.gov/nchs/nhanes/default.aspx.

## Ethics statement

Ethical review and approval was not required for the study on human participants in accordance with the local legislation and institutional requirements. The patients/participants provided their written informed consent to participate in this study.

## Author contributions

JJ and ZJ conceived and designed the study. HH and YuW accessed the data. YaZ and DC performed statistical analysis and checked date analysis process. YuZ and YaW drafted the manuscript. JJ, ZJ, and XC revised the manuscript. All authors made critical comments on the manuscript and approved the final version for submission.

## Footnotes

1
https://www.cdc.gov/nchs/nhanes/irba98.htm
