# Supplementary material for: Dietary inflammatory index, and depression and mortality risk associations in U.S. adults, with a special focus on cancer survivors
Source: Front Nutr. 2022 Dec 14;9:1034323. doi: 10.3389/fnut.2022.1034323 (PMC9795013; doi:10.3389/fnut.2022.1034323)
Supplement: Supplementary file 1 [file Table_1.DOC]

**Supplementary Table 1. Population characteristics across depression status among US subjects without cancer and cancer survivors in NHANES 2007 to 2018.**

| **Characteristic** | **Subjects without cancer** | | ***P* value** | **Subjects with Cancer** | | ***P* value** |
| --- | --- | --- | --- | --- | --- | --- |
|  | **No Depression**  **(n = 22509)** | **Depression**  **(n = 2185)** |  | **No Depression**  **(n = 2464)** | **Depression**  **(n = 276)** |  |
| **Age (years, n, %)** |  |  |  |  |  |  |
| <65 | 17869 (92.0) | 1839 (8.0) | 0.004 | 926 (88.3) | 178 (11.7) | <0.001 |
| ≥65 | 4640 (93.6) | 346 (6.4) |  | 1545 (95.1) | 104 (4.9) |  |
| **Gender (n, %)** |  |  |  |  |  |  |
| Male | 11217 (94.3) | 788 (5.7) | <0.001 | 1222 (94.2) | 86 (5.8) | 0.003 |
| Female | 11292 (90.3) | 1397 (9.7) |  | 1249 (89.9) | 196 (10.1) |  |
| **Race/ethnicity (n, %)** |  |  |  |  |  |  |
| Non-Hispanic white | 8918 (92.6) | 899 (7.4) | 0.018 | 1682 (92.7) | 161 (7.3) | <0.001 |
| Other or multi-race/ethnicity | 13591 (91.4) | 1286 (8.6) |  | 789 (86.3) | 121 (13.7) |  |
| **BMI (kg/m2, n, %)** |  |  |  |  |  |  |
| <24.9 | 6313 (93.2) | 500 (6.8) | <0.001 | 635 (92.9) | 63 (7.1) | 0.026 |
| 25 - 29.9 | 7478 (94.0) | 553 (6.0) |  | 878 (93.4) | 78 (6.6) |  |
| ≥30 | 8547 (90.0) | 1111 (10.0) |  | 932 (89.8) | 135 (10.2) |  |
| **Tobacco use (n, %)** |  |  |  |  |  |  |
| No | 13096 (94.5) | 917 (5.5) | <0.001 | 1158 (94.7) | 96 (5.3) | <0.001 |
| Yes | 9403 (89.2) | 1268 (10.8) |  | 1312 (89.2) | 186 (10.8) |  |
| **Alcohol use (n, %)** |  |  |  |  |  |  |
| No | 5300 (91.8) | 515 (8.2) | 0.169 | 599 (92.2) | 70 (7.8) | 0.787 |
| Yes | 13634 (92.4) | 1338 (7.6) |  | 1458 (91.8) | 160 (8.2) |  |
| **Co-morbidity index (times, n, %)** |  |  |  |  |  |  |
| 0 | 11024 (94.8) | 686 (5.2) | <0.001 | 558 (94.6) | 39 (5.4) | <0.001 |
| 1 | 6499 (92.5) | 578 (7.5) |  | 793 (94.1) | 64 (5.9) |  |
| 2 | 2966 (88.4) | 398 (11.6) |  | 569 (93.1) | 51 (6.9) |  |
| ≥3 | 2020 (81.0) | 523 (19.0) |  | 551 (83.2) | 128 (16.8) |  |
| **Education (n, %)** |  |  |  |  |  |  |
| <High school | 10209 (89.4) | 1304 (10.6) | <0.001 | 993 (87.1) | 160 (12.9) | <0.001 |
| ≥High school | 12283 (94.0) | 880 (6.0) |  | 1478 (94.0) | 121 (6.0) |  |
| **Marital status (n, %)** |  |  |  |  |  |  |
| Never Married | 4306 (90.7) | 480 (9.3) | <0.001 | 143 (87.5) | 33 (12.5) | <0.001 |
| Widowed / Divorced/Separated | 4473 (86.6) | 722 (13.4) |  | 782 (87.7) | 133 (12.3) |  |
| Married / Living with Partner | 13720 (94.2) | 981 (5.8) |  | 1544 (93.9) | 116 (6.1) |  |
| **Health insurance (n, %)** |  |  |  |  |  |  |
| No | 4920 (89.9) | 573 (10.1) | <0.001 | 125 (79.8) | 36 (20.2) | <0.001 |
| Yes | 17562 (92.7) | 1611 (7.3) |  | 2343 (92.5) | 244 (7.5) |  |
| **Previous cancer diagnosis (times, n, %)** |  |  |  |  |  |  |
| 1 |  |  |  | 2230 (92.1) | 244 (7.9) | 0.101 |
| ≥2 |  |  |  | 241 (88.9) | 38 (11.1) |  |
| **Ca****ncer site (n, %)** |  |  |  |  |  |  |
| Digestive system |  |  |  | 239 (88.7) | 41 (11.3) | 0.115 |
| Others |  |  |  | 2232 (92.0) | 241 (8.0) |  |
| **Duration of diagnosed (years, n, %)** |  |  |  |  |  |  |
| ≤5 |  |  |  | 1061 (92.4) | 115 (7.6) | 0.506 |
| >5 |  |  |  | 1403 (91.5) | 161 (8.5) |  |

1. The characteristics of the subjects were described as unweighted counts with weighted percentages.

**Supplementary Table 2. Subgroup analysis of the association between dietary inflammatory index score and depression status across cancer related variables in NHANES 2007 to 2018.**

|  | **Dietary inflammatory index score (quartiles)** | | | | ***P* trend** |
| --- | --- | --- | --- | --- | --- |
|  | **Q1** | **Q2** | **Q3** | **Q4** |  |
| **Previous cancer diagnosis** |  |  |  |  |  |
| 1 time |  |  |  |  |  |
| Depression (n, %) | 37 (5.6) | 36 (4.5) | 60 (7.4) | 111 (14.6) | <0.001 |
| Crude OR (95% CI) | Ref | 0.81 (0.43,1.49) | 1.36 (0.74,2.49) | 2.88 (1.87,4.76) | <0.001 |
| Adjusted OR (95% CI) | Ref | 0.70 (0.35,1.39) | 0.93 (0.45,1.18) | 1.79 (1.04,3.08) | 0.020 |
| ≥2 times |  |  |  |  |  |
| Depression (n, %) | 7 (7.0) | 7 (7.7) | 7 (9.9) | 17 (20.8) | <0.001 |
| Crude OR (95% CI) | Ref | 1.12 (0.36,3.48) | 1.47 (0.44,4.92) | 3.50 (1.00,12.25) | 0.042 |
| Adjusted OR (95% CI) | Ref | 1.66 (0.41,6.74) | 1.91 (0.60,6.11) | 1.97 (0.55,7.01) | 0.272 |
| **Cancer site** |  |  |  |  |  |
| Digestive system |  |  |  |  |  |
| Depression (n, %) | 6 (7.2) | 5 (4.9) | 6 (9.7) | 24 (20.4) | 0.010 |
| Crude OR (95% CI) | Ref | 0.69 (0.23,1.99) | 1.38 (0.41,4.65) | 3.32 (1.13,9.82) | 0.001 |
| Adjusted OR (95% CI) | Ref | 0.46 (0.13,1.66) | 2.26 (0.48,10.80) | 1.58 (0.43,5.86) | 0.123 |
| Other system |  |  |  |  |  |
| Depression (n, %) | 38 (5.6) | 38 (4.8) | 61 (7.5) | 104 (14.6) | <0.001 |
| Crude OR (95% CI) | Ref | 0.85 (0.47,1.53) | 1.37 (0.76,2.46) | 2.86 (1.72,4.76) | <0.001 |
| Adjusted OR (95% CI) | Ref | 0.79 (0.40,1.57) | 0.94 (0.47,1.86) | 1.74 (1.01,3.02) | 0.041 |
| **Duration of diagnosed** |  |  |  |  |  |
| ≤5 years |  |  |  |  |  |
| Depression (n, %) | 18 (5.2) | 17 (4.6) | 25 (7.5) | 55 (14.2) | 0.001 |
| Crude OR (95% CI) | Ref | 0.88 (0.38,2.04) | 1.49 (0.77,2.91) | 3.05 (1.46,6.34) | 0.002 |
| Adjusted OR (95% CI) | Ref | 0.92 (0.35,2.46) | 0.94 (0.41,2.17) | 1.45 (0.65,3.23) | 0.319 |
| >5 years |  |  |  |  |  |
| Depression (n, %) | 26 (6.1) | 24 (4.9) | 39 (7.5) | 72 (15.7) | <0.001 |
| Crude OR (95% CI) | Ref | 0.78 (0.34,1.79) | 1.24 (0.59,2.61) | 2.84 (1.57,5.17) | <0.001 |
| Adjusted OR (95% CI) | Ref | 0.71 (0.28,1.79) | 1.08 (0.46,2.61) | 2.12 (1.01,4.09) | 0.014 |

1. The distribution of depression were described as unweighted counts with weighted percentages.

2. Adjusted OR: adjusted for age, gender, race/ethnicity, BMI, tobacco use, co-morbidity index, education level, marital status, and health insurance status.

**Supplementary Table 3. Interaction analysis examining the interaction between dietary inflammatory index score and depression status on all-cause death, cancer-cause death and cardiovascular disease (CVD)-cause death.**

|  | All-cause death | | Cancer-cause death | | CVD-cause death | |
| --- | --- | --- | --- | --- | --- | --- |
|  | HR (95%CI) | *P* for  interaction | HR  (95%CI) | *P* for  interaction | HR (95%CI) | *P* for  interaction |
| **Subjects without cancer** |  |  |  |  |  |  |
| **Dietary inflammatory index score (quartiles)*depression** |  |  |  |  |  |  |
| Q1*no depression | - | - | - | - | - | - |
| Q2*depression | 1.17 (0.57-2.39） | 0.667 | 3.03 (0.57-16.13） | 0.193 | 0.50 (0.13-1.88） | 0.302 |
| Q3*depression | 0.90 (0.45-1.82） | 0.776 | 2.43 (0.40-14.73） | 0.336 | 0.50 (0.14-1.79） | 0.285 |
| Q4*depression | 0.91 (0.46-1.79） | 0.779 | 1.01 (0.19-5.31） | 0.986 | 0.40 (0.11-1.43） | 0.157 |
| **Subjects with Cancer** |  |  |  |  |  |  |
| **Dietary inflammatory index score (quartiles)*depression** |  |  |  |  |  |  |
| Q1*no depression | - | - | - | - | - | - |
| Q2*depression | 1.23 (0.36-4.23） | 0.742 | 2.52 (0.43-14.73） | 0.304 | 0.83 (0.07-10.57） | 0.886 |
| Q3*depression | 0.76 (0.24- 2.41） | 0.642 | 0.28 (0.04- 1.95） | 0.200 | 0.40 (0.05- 3.39） | 0.398 |
| Q4*depression | 0.44 (0.14- 1.40） | 0.164 | 0.91 (0.15- 5.50） | 0.920 | 0.56 (0.06- 4.89） | 0.603 |
